# In Silico Analysis of Pyeongwi-San Involved in Inflammatory Bowel Disease Treatment Using Network Pharmacology, Molecular Docking, and Molecular Dynamics

**DOI:** 10.3390/biom13091322

**Published:** 2023-08-28

**Authors:** Chang-Hwan Bae, Hee-Young Kim, Ji Eun Seo, Hanul Lee, Seungtae Kim

**Affiliations:** 1 Department of Korean Medical Science, School of Korean Medicine, Pusan National University, Yangsan 50612, Republic of Korea; ssaybch@gmail.com (C.-H.B.); seoji0612@naver.com (J.E.S.); ihv1432@naver.com (H.L.); 2 Korean Medicine Research Center for Healthy Aging, Pusan National University, Yangsan 50612, Republic of Korea; kimhy@pusan.ac.kr

**Keywords:** Pyeongwi-san, Ping Wei San, inflammatory bowel disease, network pharmacology, molecular docking

## Abstract

Backgound: Pyeongwi-san (PWS) is a widely used formula for treating digestive disorders in Korea and China. Inflammatory bowel disease (IBD) is characterized by progressive inflammation of the gastrointestinal tract. Emerging evidence supports the protective effect of PWS against IBD, but specific mechanisms are still elusive. Methods: Active compounds of PWS were screened from the medicinal materials and chemical compounds in Northeast Asian traditional medicine (TM-MC) in the consideration of drug-likeness and oral bioavailability. Target candidates of active compounds were predicted using the ChEMBL database. IBD-related targets were obtained from the GeneCards and DisGeNET databases. The network of composition-targets-disease was constructed. Gene ontology (GO) and Kyoto Encyclopedia of Genes and Genomes (KEGG) pathway enrichment were analyzed. Molecular docking was used to simulate the binding affinity of active compounds on target proteins and molecular dynamics was used to validate the molecular docking result. Results: A total of 26 core target proteins of PWS were related to IBD. Enrichment analysis suggested that PWS is highly associated with tumor necrosis factor signaling pathway, apoptosis, and the collapse of tight junctions. Moreover, molecular docking and molecular dynamics simulation proposed β-eudesmol and (3R,6R,7S)-1,10-bisaboladien-3-ol to ameliorate IBD through the binding to TNF and MMP9, respectively. Conclusion: Present in silico analysis revealed potential pathways and insight of PWS to regulate IBD. These results imply that the therapeutic effect of PWS might be achieved via an inhibitory effect.

## 1. Introduction

Inflammatory bowel disease (IBD), including Crohn’s disease and ulcerative colitis, is characterized by progressive intestinal inflammation. Although the incidence of IBD varies across countries, it has been increasing worldwide. The cause of IBD remains largely unknown; however, it is estimated that genetic and environmental factors are involved, and patients develop IBD in childhood or adolescence [1].

Network pharmacology is an emerging field of drug discovery that has extended insights from polypharmacology and the complexity of biology since 2007 [2]. Through computational methodology, network pharmacology can provide information, such as protein-protein interactions, the association of traditional medicine composition-target-disease (CTD) networks, and viable therapeutic target pathway enrichment of interesting drugs or diseases. Since traditional formulae in traditional Korean medicine are used in combination with various medicinal substances, network pharmacology from a holistic perspective plays an important role in understanding the effects and mechanisms of herbal medicines. For example, using network pharmacological analysis, Zhou and Wu showed that the mitogen-activated protein kinase pathway is a potential pharmacological pathway of Eucommiae cortex against osteoporosis [3], and Dou et al. reported the protective effect of tanshinone II in *Salvia miltiorrhiza* Bunge in an acute kidney injury model through pregnane X receptor activation [4].

Pyeongwi-san (PWS, Ping Wei San in Chinese), which was first described in Prescriptions from the Great Peace Imperial Grace Pharmacy (*Taipinghuiminhejijufang* in Chinese) during the Song dynasty, is a traditional formula consisting of Atractylodis Rhizoma (ATR), Citri Unshius Pericarpium (CUP), Glycyrrhizae Radix et Rhizoma (GR), Magnoliae Cortex (MC), Zizyphi Fructus (ZF), and Zingiberis Rhizoma Recens (ZR). PWS is widely used for the treatment of various digestive disorders including dyspepsia, nausea, abdominal pain, and anorexia [5]; it is most frequently used for the treatment of functional dyspepsia in Korea [6]. Studies have shown that PWS has anti-inflammatory effects in dextran sulfate sodium and *Clostridium difficile*-induced IBD mouse models via restoration of mucus thickness, tight junctions, and microbiota ratio [7] and in lipopolysaccharide-challenged RAW264.7 cells by decreasing prostaglandin E2, nitric oxide, interleukin 6, and tumor necrosis factor (TNF) alpha [8]; however, the specific mechanism of PWS in IBD is unclear. In this study, the association between the target proteins and active compounds of PWS against IBD was analyzed using network pharmacology, molecular docking, and molecular dynamics simulation.

## 2. Materials and Methods

### 2.1. Active Compounds Screening

The compound lists of PWS were retrieved from a database of medicinal materials and chemical compounds in Northeast Asian traditional medicine (TM-MC; https://informatics.kiom.re.kr/compound/ accessed on 3 October 2022) [9]. The simplified molecular input line entry system (SMILES) for each compound was obtained and verified using the PubChem database (https://pubchem.ncbi.nlm.nih.gov/ accessed on 24 November 2022) for further analysis. If isomeric SMILES of compounds were not included in the PubChem database, canonical SMILES were selected for analysis. Human intestinal absorbable compounds and those negative for P-glycoprotein substrates were classified using SwissADME (http://www.swissadme.ch/index.php/ accessed on 28 November 2022) based on SMILES within 200 characters. The remaining compounds that were filtered by no violation of Lipinski’s “rule of five” and predicted to have over 20% oral bioavailability were considered active compounds [10,11].

### 2.2. Disease-Related Gene Selection

IBD-related genes were queried using the GeneCards (https://www.genecards.org/ accessed on 12 October 2022) and DisGeNET (https://www.disgenet.org/ accessed on 12 October 2022) databases [12,13]. The top 10% of protein-coding genes from GeneCards and genes sorted by an evidence index over 0.7 from DisGeNET were selected to maintain high disease gene association.

### 2.3. Target Prediction

Target proteins from active compounds of PWS were predicted in the model provided by version 30 of ChEMBL database (https://www.ebi.ac.uk/chembl/ accessed on 29 November 2022) [14,15]. SMILES of compounds was queried into ChEMBL multitask neural network model (https://github.com/chembl/chembl_multitask_model accessed on 29 November 2022), and target proteins were filtered by the probability over 0.7 and species for human.

### 2.4. Network Construction

To identify the association among active compounds, target candidates, and disease, the CTD network was constructed. Core protein-protein interaction was also extracted using Cytoscape 3.9.6 software (Boston, MA, USA) [16]. Important nodes of the protein-protein interaction were selected by the degree centrality and eigenvector centrality over third quartile.

### 2.5. Gene Ontology (GO) and Kyoto Encyclopedia of Genes and Genomes (KEGG) Pathway Analysis

GO and KEGG pathways were investigated to understand the contribution of PWS to the functionality and underlying pathways [17,18]. ShinyGO version 0.77 (http://bioinformatics.sdstate.edu/go/ accessed on 6 December 2022) was used to analyze GO and KEGG pathways [19]. The statistical cutoff threshold was a *p*-value of 0.5. The top 20 results of GO and the top 30 results of the KEGG pathway were plotted in accordance with the fold enrichment, reflecting the false discovery rate and number of genes.

### 2.6. Molecular Docking

The crystal structure of the target protein was acquired from the RCSB Protein Data Bank (https://www.rcsb.org/ accessed on 20 December 2022), and the 3D structure of each ligand was prepared from the PubChem database. Heteroatoms including ions and crystal waters were discarded before docking simulation. Ligand file was converted into autodock compatible file format (.sdf file to .pdb file format) via PyMOL software (Schrödinger LLC., New York, NY, USA). Ligand was prepared to recognize rotational bonds, charge, and unnecessary hydrogen atoms. Protein was prepared by processing charge, adding hydrogen atoms then merging non-polar hydrogen for docking. The binding site searching grid box was set to cover all protein surfaces. Ten independent docking simulations for each ligand were performed to avoid bias. The exhaustiveness of the simulation was set to 32. All docking simulations were performed using AutoDock Tools version 1.5.7 and AutoDock Vina version 1.2.3 [20,21,22]. The pose with the lowest binding affinity (kcal/mol) between the active compound and protein was considered a stable protein-ligand binding pose. Data were presented using Pymol version 2.5 and LigPlot+ version 2.2software [23].

### 2.7. Molecular Dynamics Simulation

Molecular dynamics simulation was performed using GROMACS software version 2023.1 [24]. Prior to the simulation, each protein structure was prepared by discarding crystal water molecules and heteroatoms from a coordinate file. In particular, only chain A of MMP9 was used to optimize simulation computing power. In addition, broken sequences in chain B and chain C of TNF were filled with the sequence of another TNF (RCSB protein databank ID: 5YOY) as a template using modeler software version 10.4 [25].The coordinates of the active compounds were selected from the most stable binding pose of the most stable molecular docking simulation. The coordinates and parameters of each active compound for MD were prepared under the Charmm36 force field [26]. TIP3P explicit water model was applied for solvation in dodecahedron periodic boundary conditions. Sodium and chloride ions were added by 0.154 M to match the physiological condition. Additional sodium ions were added to neutralize the system. Then, the energy of the system was minimized. Berensden thermostat and Parinello–Rahman barometer were used for NVT and NPT equilibration, respectively. Long-range electrostatics were set by the particle mesh Edward method [27]. Searching neighboring cells was set by the Verlet scheme. After simulation for 50 ns, root mean square of the deviation (RMSD) and root mean square of the fluctuation (RMSF) were evaluated. Hydrogen bond interactions during simulation were assessed using VMD software [28]. The distance cutoff and angle for hydrogen bonds were set to 3.5 nm and 30 degrees, respectively. The results were visualized using PyMol and software.

## 3. Results

### 3.1. Screening Active Compounds of PWS

**Figure 1:**
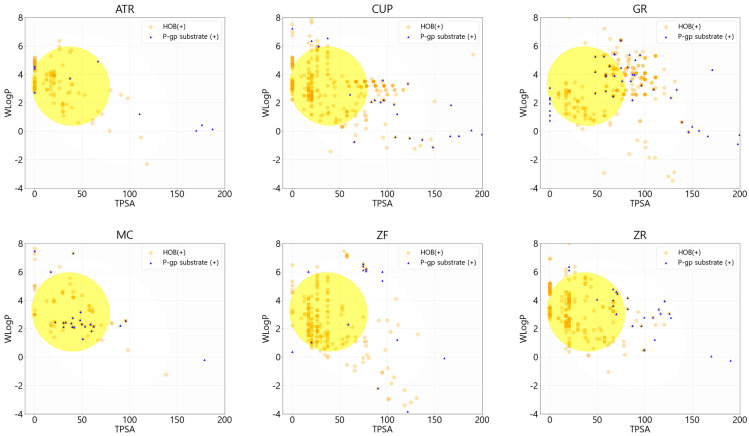
BOILED-Egg and P-glycoprotein substrate prediction plots of individual medicinal herbs of Pyeongwi-san. Compounds within white oval area represent human intestinal absorbable. Especially, compounds with in yellow area are also permeable to brain-blood barrier. Orange dots represent negative substrates for P-glycoprotein, and blue triangles are positive substrates for P-glycoprotein which is expected to excrete from cells. TPSA, topological polar surface area; ATR, Atractylodis Rhizoma; CUP, Citri Unshius Pericarpium; GR, Glycyrrhizae Radix et Rhizoma; MC, Magnoliae Cortex; ZF, Zizyphi Fructus; ZR, Zingiberis Rhizoma Recens.

**Figure 2:**
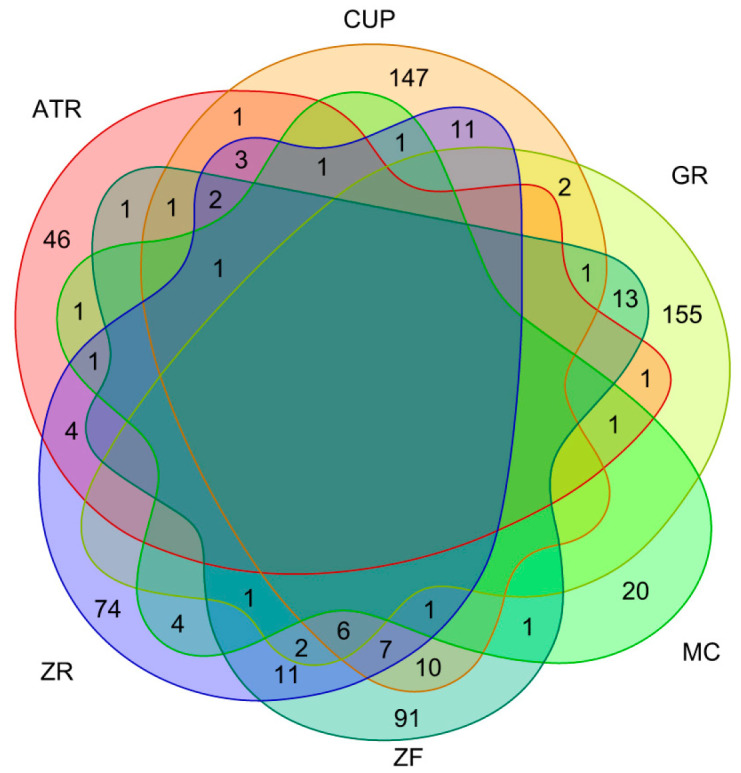
Venn diagram of compounds in traditional medicines. All the numbers represent active compounds that passed criteria of drug-likeness and oral-bioavailability. ATR, Atractylodis Rhizoma; CUP, Citri Unshius Pericarpium; GR, Glycyrrhizae Radix et Rhizoma; MC, Magnoliae Cortex; ZF, Zizyphi Fructus; ZR, Zingiberis Rhizoma Recens.

**Figure 3:**
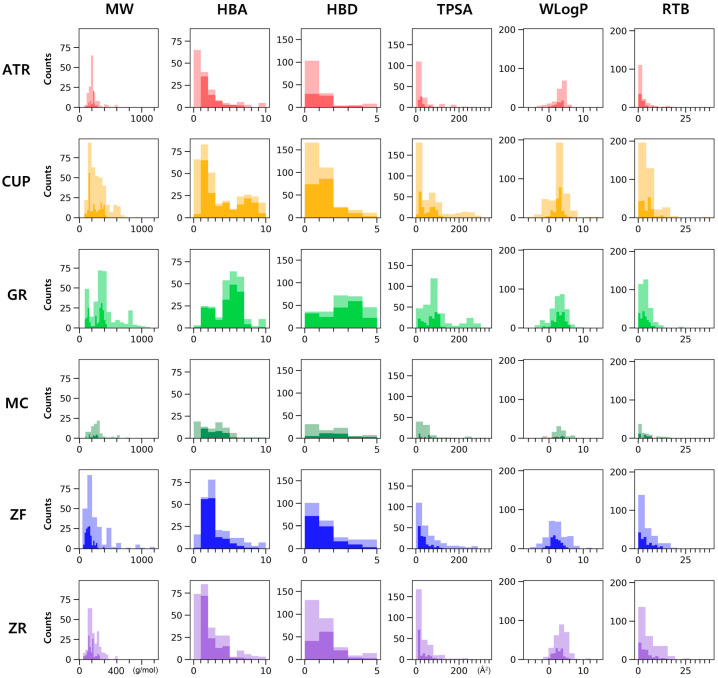
Histogram of physicochemical properties of active compounds in each composition of PWS. Light-colored histogram represents distribution of all the compounds in the composition, and darker histogram shows distribution of active compounds that passed drug-likeness and oral-bioavailability criteria. MW: molecular weight, HBA: H-bond acceptor, HBD: H-bond donor, TPSA: topological polar surface area, RTB: rotatable bond. ATR, Atractylodis Rhizoma; CUP, Citri Unshius Pericarpium; GR, Glycyrrhizae Radix et Rhizoma; MC, Magnoliae Cortex; ZF, Zizyphi Fructus; ZR, Zingiberis Rhizoma Recens.

We investigated the human intestinal absorption of individual compounds of PWS by two physicochemical properties, topological polar surface area (TPSA) and lipophilicity, using the log P method developed by Wildman and Crippen (WLogP) based on the BOILED-Egg model (Figure 1) [29]. The filtered compound list was narrowed to ensure pharmacological compatibility for further analysis. Within criteria of no violation of the “rule of five” and negative substrate for P-glycoprotein, after exclusion of prediction results of human oral bioavailability below 20%, 64 of 161 compounds for ATR, 195 of 379 compounds for CUP, 182 of 320 compounds for GR, 33 of 89 compounds for MC, 149 of 257 compounds for ZF, and 130 of 281 compounds for ZR remained. Excluding duplicates, the remaining 622 compounds were considered active compounds (Figure 2). The light shadow histogram shows all compounds in the composition, and the darker histogram represents the distribution of physicochemical properties of the remaining 622 compounds that have passed high drug-likeness and oral-bioavailability criteria (Figure 3). The whole list of active compounds was provided in Table S1.

### 3.2. Target Prediction

**Figure 4:**
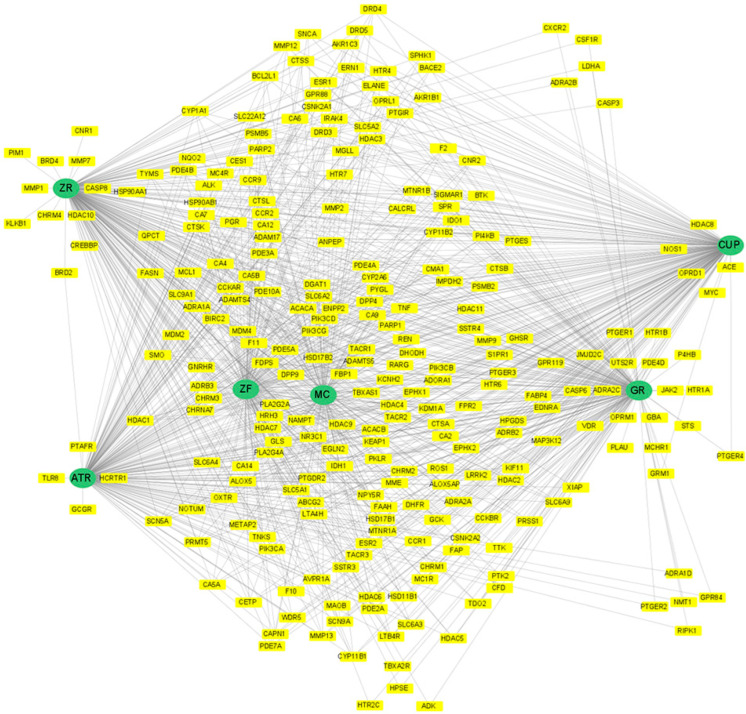
Network of composition and target proteins. Green circle represents composition, and yellow box represents target proteins. ATR, Atractylodis Rhizoma; CUP, Citri Unshius Pericarpium; GR, Glycyrrhizae Radix et Rhizoma; MC, Magnoliae Cortex; ZF, Zizyphi Fructus; ZR, Zingiberis Rhizoma Recens.

Candidate target proteins of each compound were predicted using version 30 model of the ChEMBL database. SMILES of the compound were queried, and a total of of 290 targets have a probability over 0.7. After discarding duplicates and leaving those related to Homo sapiens, 257 target candidates of PWS remained (Figure 4, Table S1). Composition-compound-target protein pairs were also presented in Table S1.

### 3.3. Disease Gene Association

**Figure 5:**
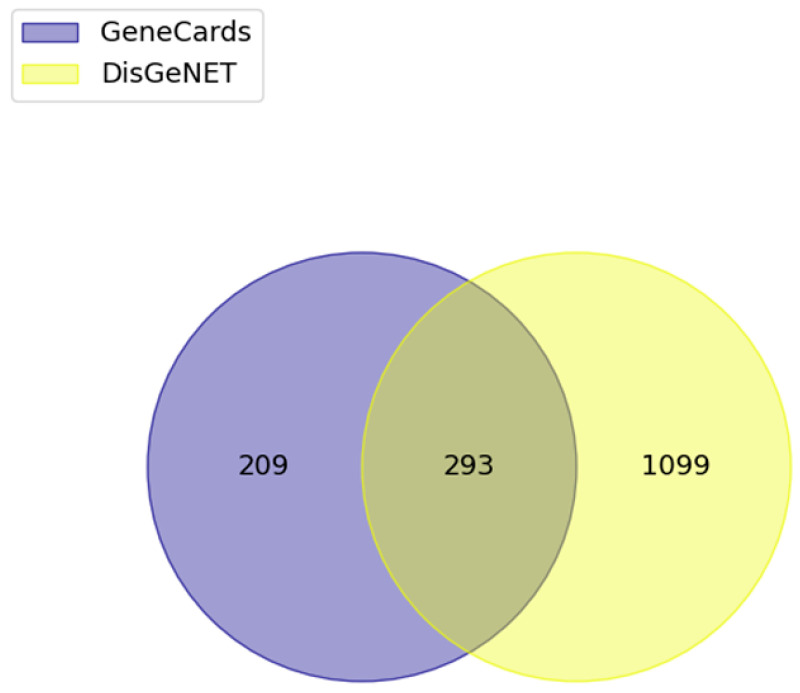
Venn diagram of the intersection of IBD related targets. Top 10% protein coding genes in the GeneCards and target genes of evidence index over 0.7 in the DisGeNET were selected.

We queried “inflammatory bowel disease” into the DisGeNET and GeneCards databases to identify disease-associated genes. As a result, 1391 IBD-associated genes were collected by filtering with over 0.7 of evidence index in the DisGeNET database. In the GeneCards database, 502 IBD-related protein-coding genes in the top 10% of the score are obtained. Consequently, 293 targets were overlapped and used for further analysis (Figure 5).

### 3.4. Network Analysis

**Figure 6:**
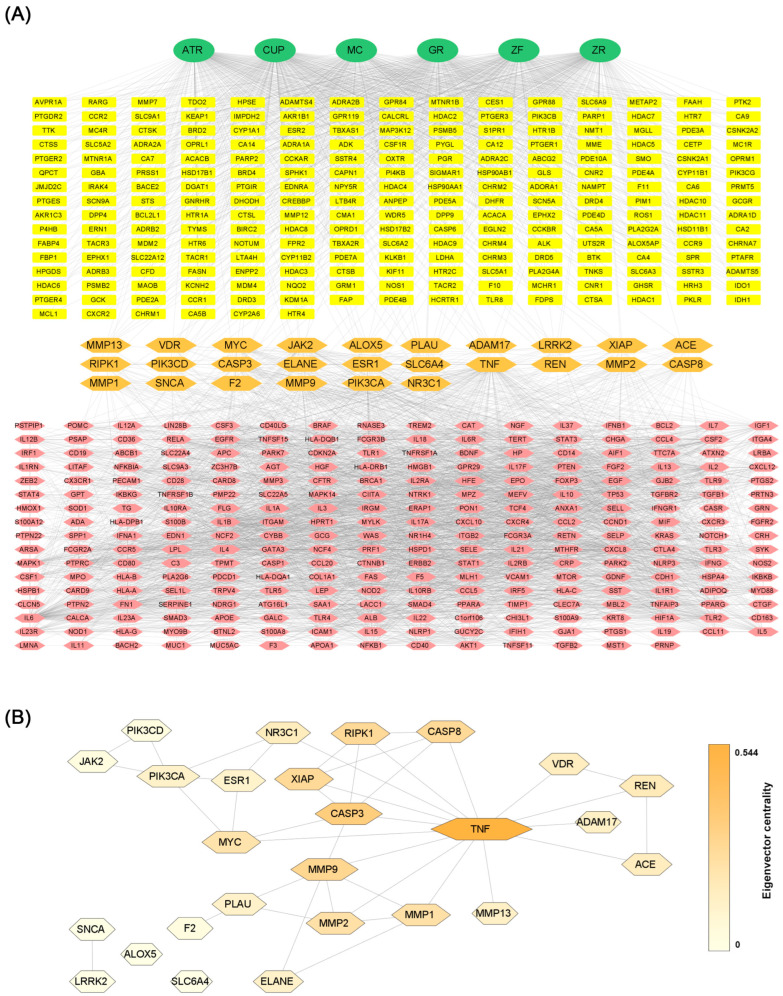
(**A**) Composition-target-disease network between PWS and IBD. Green circle, yellow box, and pink hexagon represent composition of PWS, targets of PWS, and IBD-related targets, respectively. Orange hexagon shows potential targets of PWS involved in the pathogenesis of IBD. (**B**) Protein-protein interaction of 26 candidate target proteins of PWS involved in IBD from panel A. Size and color of node represent the degree and eigenvector centrality. ATR, Atractylodis Rhizoma; CUP, Citri Unshius Pericarpium; GR, Glycyrrhizae Radix et Rhizoma; MC, Magnoliae Cortex; ZF, Zizyphi Fructus; ZR, Zingiberis Rhizoma Recens.

The CTD network between PWS and IBD was constructed using Cytoscape software based on the STRING database (Figure 6A). Centralities of the network were analyzed by the CytoNCA plugin in Cytoscape (Table 1) [30]. The protein-protein interaction network overlapped between the target of PWS and IBD has 26 nodes and 41 edges (Figure 6B). With respect to degree centrality, tumor necrosis factor (TNF) showed the highest degree of centrality at 14, followed by caspase 3 (CASP3), and matrix metalloproteinase (MMP9), and phosphatidylinositol-4,5-bisphosphate 3-kinase catalytic subunit alpha. With respect to eigenvector centrality, which reflects the centrality weight of the neighbor node, TNF, CASP3, MMP9, and followed by X-linked inhibitor of apoptosis, caspase 8, and receptor-interacting serine/threonine kinase 1 showed relatively high eigenvector centrality. Specifically, we filtered core target proteins by the degree and eigenvector centralities over the 75th quartile. The third quartile value of the degree centrality is 4, and the eigenvector centrality is about 0.217. Then, TNF (degree centrality: 14, eigenvector centrality: 0.544), CASP3 (degree centrality: 6, eigenvector centrality: 0.353), and MMP9 (degree centrality: 6, eigenvector centrality: 0.289) remained (Table 1).

### 3.5. GO and KEGG Pathway Enrichment Analysis

**Figure 7:**
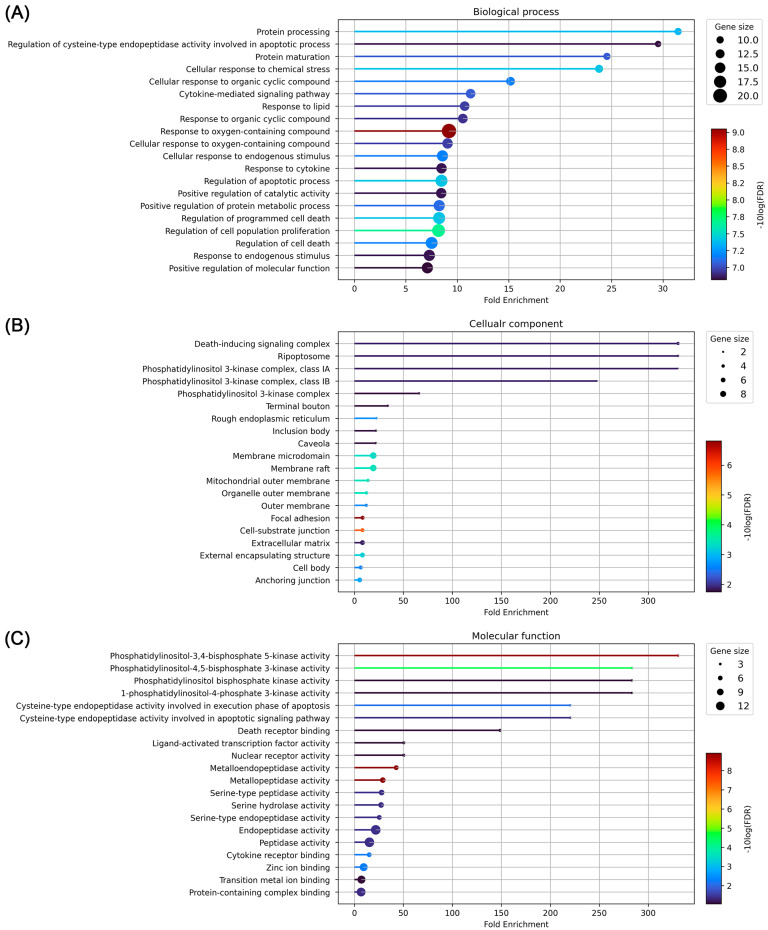
Gene ontology enrichment analysis. (**A**) Biological process, (**B**) cellular composition, (**C**) molecular function. Data were sorted in descending order by fold enrichment. Bubble size represents how many genes are involved in (FDR cutoff < 0.05).

**Figure 8:**
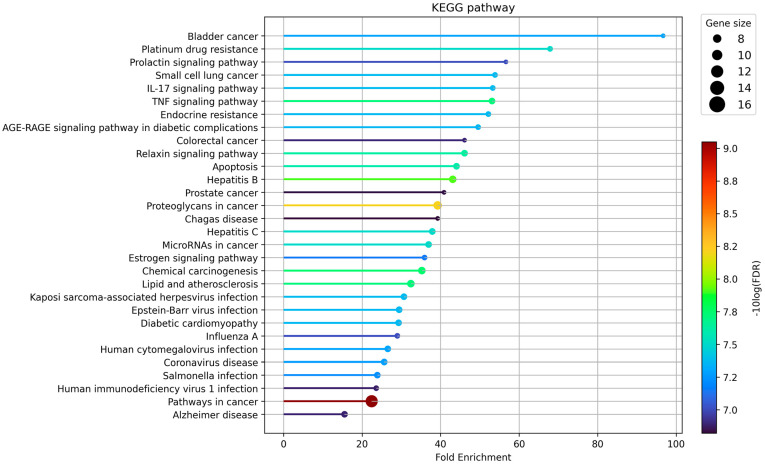
Top 30 pathways of Kyoto Encyclopedia of Genes and Genomes enrichment analysis. Data were sorted in descending order by fold enrichment. Bubble size represents how many genes are involved (FDR cutoff < 0.05).

GO enrichment was analyzed to investigate the biological processes of 26 overlapping target candidates of PWS involved in IBD. We sorted the GO analysis results with a low false discovery rate and then high fold enrichment. The results showed that protein processing (GO:0016485), regulation of cysteine-type endopeptidase activity involved in the apoptotic process (GO:0043281), protein maturation (GO:0051604), cellular response to chemical stress (GO:0062197), and cellular response to organic cyclic compound (GO:0071407) were enriched in biological processes. In the case of cellular components, death-inducing signaling complex (GO:0031264), ripoptosome (GO:0097342), phosphatidylinositol 3-kinase complex, class IA (GO:0005943), phosphatidylinositol 3-kinase complex, class IB (GO:0005944), and phosphatidylinositol 3-kinase complex (GO:0005942) were predicted as candidate locations. Phosphatidylinositol-3,4-bisphosphate 5-kinase activity (GO:0052812), 1-phosphatidylinositol-4-phosphate 3-kinase activity (GO:0035005), phosphatidylinositol-4,5-bisphosphate 3-kinase activity (GO:0046934), phosphatidylinositol bisphosphate kinase activity (GO:0052813), and cysteine-type endopeptidase activity involved in the apoptotic signaling pathway (GO:0097199) were enriched in molecular function (Figure 7). We further investigated which biological pathway was enriched in the KEGG database to elucidate the possible mechanisms. The results showed that bladder cancer (hsa:05219), platinum drug resistance (hsa:01524), prolactin signaling pathway (hsa:04917), small cell lung cancer (hsa:05222), IL-17 signaling pathway (hsa:04657), and TNF signaling pathway (hsa:04668) were highly enriched (Figure 8). If excluding unrelated diseases to IBD, the IL-17 signaling pathway, TNF signaling pathway, and apoptosis (hsa:04210) are highly enriched. Collectively, TNF signaling and apoptosis-related proteins can be suggested as potential candidates of PWS regulating IBD.

### 3.6. Molecular Docking Simulation

**Figure 9:**
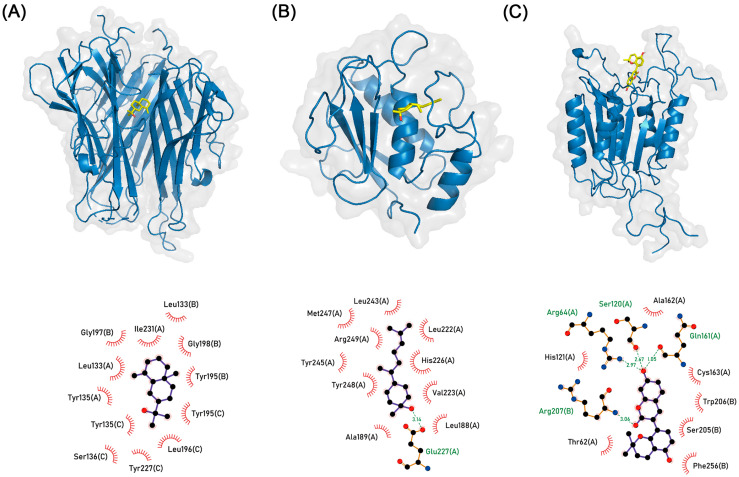
Representative stable binding pose of molecular docking simulation. Upper panel shows the docking poses of ligands (in yellow) and proteins (in blue cartoon), as well as the molecular surface of proteins (in light grey). Lower panel shows molecular interactions between ligands and proteins, presenting the residue of the proteins and the involved chains in the interaction. Hydrogen bond presents as a green dashed line and distance (angstrom). (**A**) β-eudesmol to TNF, (**B**) (3R,6R,7S)-1,10-bisaboladien-3-ol to MMP9, and (**C**) glabrocoumarin to CASP3.

In accordance with network and enrichment analysis which points to TNF, CASP3, and MMP9 as pivotal elements; we simulated molecular docking to find whether active compounds of PWS stably bind to the core target protein. Each ligand binding pocket of proteins was determined using the Protein Data Bank database and literature. We independently simulated the molecular docking of active compounds that target TNF, CASP3, and MMP9 ten times. The results of most simulations exhibited a stable binding pose consistent with already-known inhibitors. Generally, a pose with the lowest binding affinity or under −6 kcal/mol is regarded as stable binding. Molecular docking simulations almost showed a binding pose with binding affinity below −6 kcal/mol (Table S2). Among them, the lowest binding affinity of TNF (PDB ID: 7JRA) to β-eudesmol from ZR is −8.259 kcal/mol, CASP3 (PDB ID: 2C2M) to glabrocoumarin from GR is −8.238 kcal/mol, and MMP9 (PDB ID: 4WZV) to (3R,6R,7S)-1,10-bisaboladien-3-ol from ZR is −8.131 kcal/mol, respectively (Figure 9, Table 2). Also, a list of other binding pose candidates is presented in Table S2.

### 3.7. Molecular Dynamics Simulation

A molecular dynamics simulation is performed to verify the result of molecular docking using GROMACS with the Charmm36 force field. Prior to the simulation, unmodeled sequences that Pro182 to Ala187 (Pro-Glu-Gly-Ala-Glu-Ala) in the chain of B and Thr181 to Ala187 (Thr-Pro-Glu-Gly-Ala-Glu-Ala) in the chain C of TNF were filled with the template sequence of another TNF structure using the Modeller software of version 10.4. Without this procedure, the generation coordinate of the protein for the molecular dynamics would have failed.

**Figure 10:**
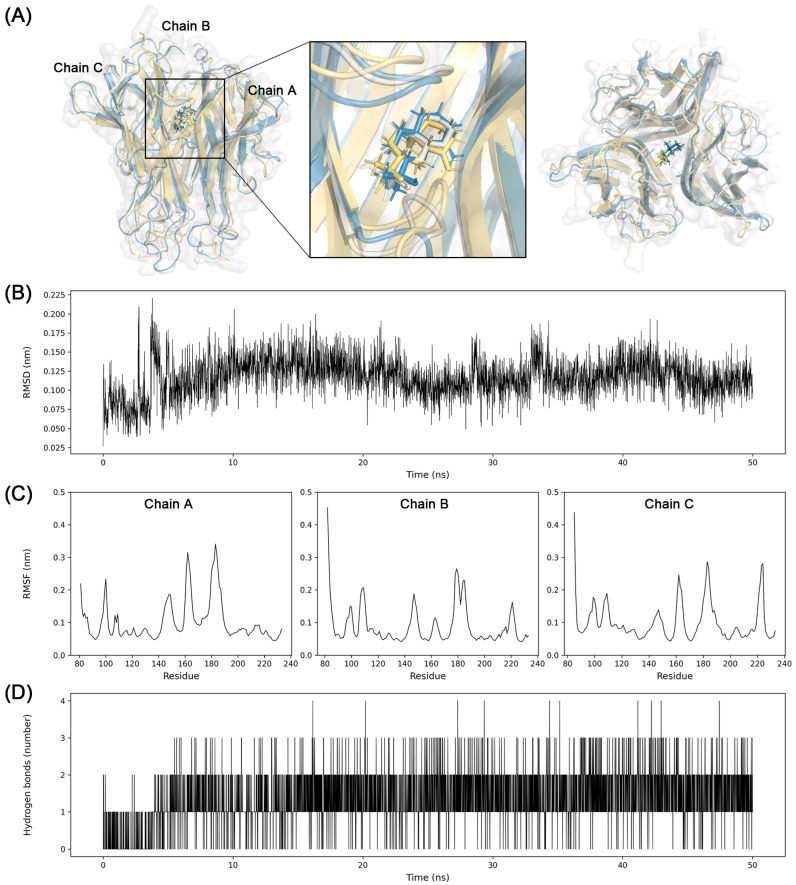
Molecular dynamics simulation analysis for β-eudesmol binding to TNF trimer. (**A**) Three snapshots of conformations during the simulation were taken at 25 ns intervals and merged (yellow, 0 ns; white, 25 ns; blue, 50 ns). Molecular surface of TNF at 50 ns was represented as white. (**B**) RMSD between β-eudesmol and TNF, (**C**) RMSF of TNF, and (**D**) hydrogen bond interactions between β-eudesmol and TNF were presented.

The simulation result of TNF showed stable binding of β-eudesmol to protein during 50 ns. The average RMSD of the distance between β-eudesmol and TNF was 0.11 nm with a standard deviation of 0.02 nm. Three snapshots were taken at 25 ns intervals to understand the trajectory of ligand and protein. β-eudesmol exhibited stability in the cavity between trimer of TNF during the simulation. The stably maintained hydrogen bond interactions also supported the binding pose of β-eudesmol to TNF (Figure 10).

**Figure 11:**
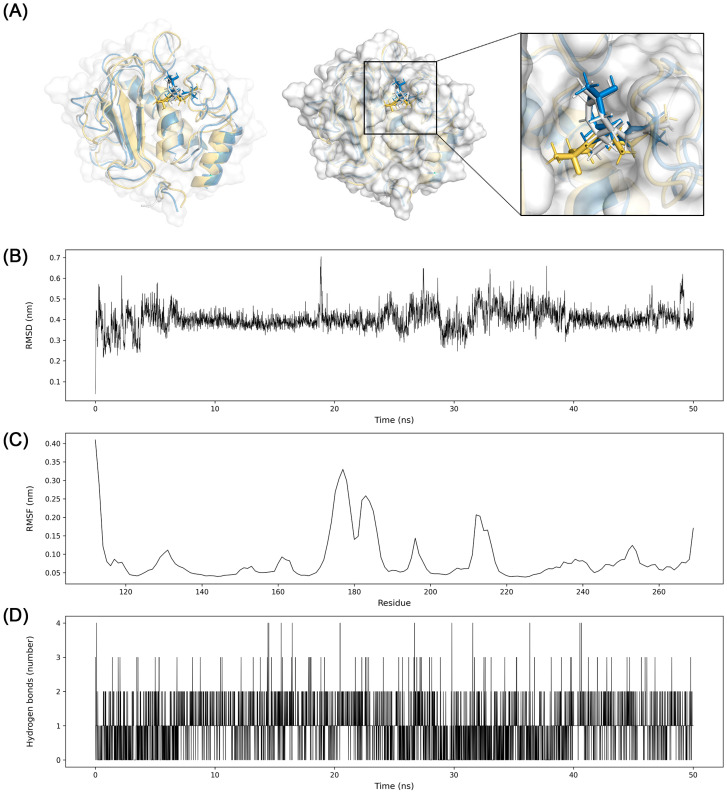
Molecular dynamics simulation analysis for (3R,6R,7S)-1,10-bisaboladien-3-ol binding to MMP9. (**A**) Three snapshots of conformations during the simulation were taken at 25 ns intervals and merged (yellow, 0 ns; white, 25 ns; blue, 50 ns). Molecular surface of MMP9 at 50 ns was represented as white. (**B**) RMSD between (3R,6R,7S)-1,10-bisaboladien-3-ol and MMP9, (**C**) RMSF of MMP9, and (**D**) hydrogen bond interactions between (3R,6R,7S)-1,10-bisaboladien-3-ol and MMP9 were presented.

The binding pose of (3R,6R,7S)-1,10-bisaboladien-3-ol to MMP9 was stably maintained in the active site. Specifically, the planar conformation was bent quickly, but the structure binding to the pocket structure binding to the pocket next to the active site kept position throughout the simulation. The RMSD of the distance was 0.4 nm with the standard deviation of 0.05 nm. Hydrogen bond interactions were also stably maintained (Figure 11).

**Figure 12:**
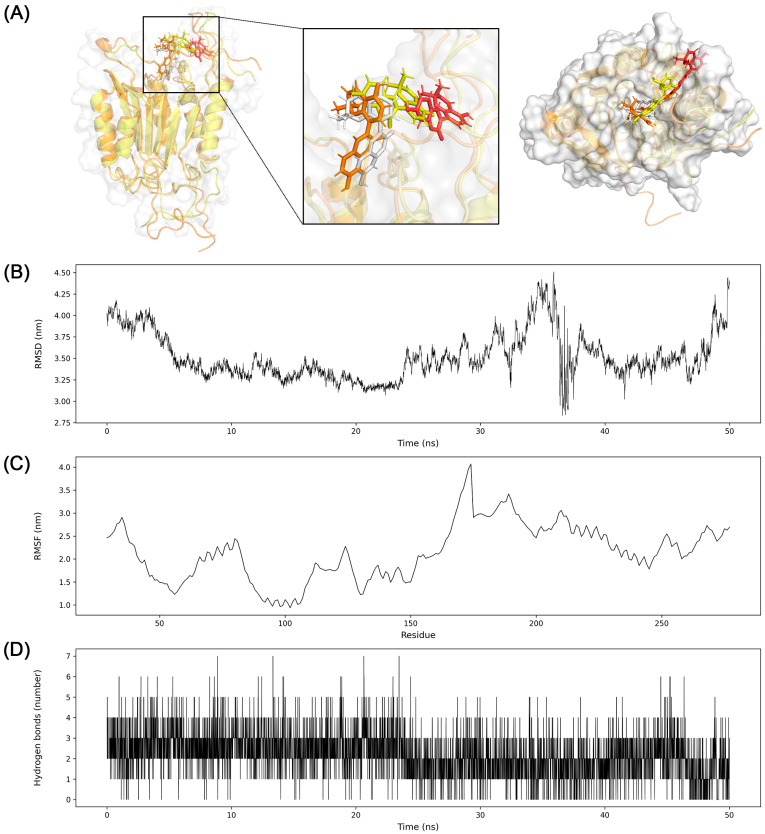
Molecular dynamics simulation analysis for glabrocoumarin to CASP3. (**A**) Four snapshots of conformations during the simulation were taken and merged (orange, 0 ns; white, 25 ns; yellow, 36 ns; red, 50 ns). Gloabrocoumarin has moved apart from the initial binding site in the catalytic domain during simulation. Molecular surface of CASP3 at 50 ns was represented as white. (**B**) RMSD between glabrocoumarin and CASP3, (**C**) RMSF of CASP3, and (**D**) hydrogen bond interactions between glabrocoumarin and CASP3 were presented.

However, the MD simulation of glabrocoumarin to CASP3 exhibited a different result from the molecular docking. The average RMSD was 3.5 nm with a standard deviation of 0.27 nm. The RMSD of glabrocoumarin to CASP3 was approximately 4 nm at the beginning of the simulation, followed by a decrease to 3.1 nm around 20 ns. Then RMSD fluctuated rapidly around 36 ns, and increased until the end of the simulation. Glabrocoumarin kept a binding position from the beginning of the simulation until about 25 ns, and slightly moved away from the catalytic domain (Figure 12).

## 4. Discussion

In the present study, we investigated the possible therapeutic targets and pathways of PWS involved in IBD using network pharmacology. Prior to analysis, metadata of PWS are required to identify the physicochemical properties of compounds contained in each herbal medicine. There are several databases of systemically organized information about traditional medicine, including TM-MC, traditional Chinese medicine integrated database, TCM database@taiwan, and traditional Chinese medicine systems pharmacology database analysis platform [9,31,32,33]. In this study, we chose TM-MC as the fundamental database for the following reasons. First, TM-MC contains information on the practical traditional medicines used in Northeast Asia. Second, a database based on the latest extensive literature was curated by experts, including biologists and doctors of Korean medicine. Third, the TM-MC presented the source of information that led to a clear determination of knowledge.

To screen therapeutic compound candidates, drug-likeness and oral bioavailability must be considered. The definition of drug-likeness varies depending on the literature; nevertheless, therapeutic effects are expected in compounds with high drug-likeness in general. Oral bioavailability refers to the ratio of the amount that reaches the systemic circulation after oral administration of a drug [34].

Lipinski pointed out that a compound with poor absorption and permeation can be considered as low drug-likeness because it tends to fail early clinical trials [11]. In his literature, Lipinski suggests criteria named “the rule of five”. It contains four guidelines for chemical compounds to avoid low drug-likeness as follows: molecular weight over 500, the number of hydrogen bond donors over 5, the number of hydrogen bond acceptors over 10, calculated Log P over 5 [11]. Despite several approved drugs that violate the “rule of 5” such as antibiotics and antifungals, it is still widely accepted as a rule to predict drug-likeness of compounds. Similarly, Veber showed that good oral bioavailability is related to the molecular properties with less than 10 rotatable bonds and polar surface area under 140 Ǻ^2^ [35]. BOILED-egg is a predictive model for determining human intestinal absorption and brain-blood permeability. This model was developed based on datasets including experimental results [36]. It can be calculated using two physicochemical properties: TPSA and WLogP [29]. In particular, the human intestinal absorption classification of this supervised machine-learning model was reliable enough that it showed 10-fold cross-validation with a Matthews correlation coefficient of 0.6531 and an accuracy of 0.9156 [29]. Based on the BOILED-egg model, 558 of the 1660 compounds predicted to not be absorbed into the human intestine were excluded. The remaining compounds were also investigated for their potential as substrates for P-glycoprotein because P-glycoprotein protects the human body from exogenous toxicity by excreting toxic molecules and their metabolites [37]. As a result, 120 of the 1102 compounds that are predicted as substrates for p-glycoprotein were excluded. “The rule of five” was applied to screen compounds with high drug-likeness. During this step, 64 of 982 compounds were excluded. Then, the oral bioavailability with a cutoff of 20% was predicted using Wei’s method [10]. This model is built based on the dataset including 1142 training and 287 test molecules, with good performance scores. It is noteworthy that oral bioavailability is shown to be negatively correlated with topological descriptors among the more than 1000 molecular descriptors [10,38]. As a result, 72 of 918 compounds were excluded. Finally, excluding duplicates, 622 compounds were screened and considered as active compounds of PWS. Only the rule-based method cannot overwhelm the limitations for screening drugs until now. Consequently, we determined therapeutic candidates according to the criteria mentioned above using a combination of rule-based and machine-learning methods, which can be expected to have drug-likeness and high oral bioavailability in silico. Figure 3 shows the physicochemical property histogram of active compounds versus all compounds in the PWS. A darker histogram refers to active compounds, and a lighter refers to all compounds. Distribution difference indicates that active compounds are not simply determined by a rule-based method. Next, IBD-related genes are searched from GeneCards and DisGeNET databases. The top 10% genes in the search result of GeneCards and the genes from DisGeNET with the evidence index over 0.7 to maintain high association were selected, and their common genes were used for further investigation.

The CTD network was constructed from the associations between predicted targets of active compounds of PWS and IBD-related targets. Overlapped targets between PWS and IBD in the network suggest that PWS is involved in IBD pathogenesis. A total of 26 pivotal candidates were derived from the network, and protein-protein interactions were investigated. Topological network analysis, which can be used for drug repositioning, provides a novel clue to the underlying biological complexity [39]. Centrality measurement is an approach used in topological network analysis. Degree centrality is a measure of the number of interactions of a node. Similarly, eigenvector centrality refers to centrality that reflects the weight of a node [40]. Betweenness centrality refers to the impact of a particular node connecting nodes that are not directly connected, and closeness centrality is a measure of how close a node is to all other nodes in a network. Although the betweenness and closeness centralities are also common measurements to analyze biological networks, interpretation in a biological context is required. MYC1 has a fairly high betweenness centrality of 75.7 (Table 1), which implies that CASP3 and PIK3CA may work together through MYC to regulate IBD (Figure 6B). In this study, we chose node centrality and eigenvector centrality to evaluate the impact of nodes purely in this consideration. Nevertheless, we also provide betweenness and closeness centralities for implications beyond this study (Table 1).

TNF, CASP3, and MMP9 showed high centrality in both degree centrality and eigenvector centrality. TNF is implicated in the inflammatory response. Infliximab, a monoclonal antibody against TNF, is used to treat inflammatory diseases [41]. The anti-TNF strategy also restored the microbiota composition of patients with Crohn’s disease to a healthy state [42]. CASP3 plays a major role in apoptosis. CASP3 induces apoptosis by regulating the destruction of cellular structures, whereas XIAP suppresses apoptosis by inhibiting CASP9, which is directly involved in the activation of CASP3 [43]. Increased expression of active CASP3 has been observed in the colon tissue of patients with Crohn’s disease and in a 2,4,6-trinitrobenzene sulfonic acid-induced colitis animal model [44,45]. MMP is known to disrupt intestinal conditions in IBD. An increase in MMP9 expression is observed in patients with IBD and leads to the activation of myosin light chain kinase, which weakens tight junction permeability [46,47]. Molecular docking is a computational method for determining the binding pose of a ligand to a target protein. In this study, docking simulation showed stable binding affinity of active compounds in PWS with core target proteins TNF, CASP3, and MMP9. Recent research pointed out that the binding affinity of compound to protein under −5 kcal/mol exhibits the stable binding [48,49]. In this research, binding affinities of other active compounds were observed under −7 kcal/mol. They might be potential candidates to interact with their target proteins (Table S2).

Molecular dynamics simulation is the computational method that tracks the trajectory of the molecules based on molecular mechanics [50]. Although molecular docking analyzes massive interactions of ligands and receptors at high-throughput speed, the static state cannot entirely explain the dynamics of the system. Therefore, we further analyzed molecular dynamics to verify the results of molecular docking.

As a result, β-eudesmol stably binds to the central cavity of the TNF trimer (Figure 10). Around 2 hydrogen bond interactions were sustained during the simulation. The trajectory and stable RMSD suggest that the β-eudesmol firmly binds to TNF. The binding position of β-eudesmol is similar to the previous research [51]. The binding of the inhibitor to TNF changes the conformation of TNF trimer to asymmetric so as to prevent interactions with the TNF receptor [52]. The substrate binding pocket S1′ of MMP9 is comprised of Asp185 to Leu188 and Pro246 to Tyr248. This pocket could interact with various substrates and inhibitors by hydrogen bonds [53]. In this study, the conformation of (3R,6R,7S)-1,10-bisaboladien-3-ol was slightly changed during the simulation; however, the dynamics and snapshot of (3R,6R,7S)-1,10-bisaboladien-3-ol showed a stable binding pose in the cavity composed of Leu222, Val223, His226, Tyr245, Met247, and Arg249(Figure 11).

Interestingly, the analysis of the molecular dynamics of glabrocoumarin to CASP3 showed a different implication than the result of molecular docking. Molecular docking simulation suggested the binding pose of glabrocoumarin to CASP3 as stable with the binding affinity of −8.131 kcal/mol. Nevertheless, molecular dynamics provided the trajectory that glabrocoumarin moves apart from the initial binding pose during the simulation around 37 ns (Figure 12).

In summary, the inhibitory effects of β-eudesmol to TNF and (3R,6R,7S)-1,10-bisaboladien-3-ol to MMP9 were suggested by molecular docking and molecular dynamics simulation. These results also imply that the analysis of the binding of small molecules to proteins should be interpreted using molecular docking and molecular dynamics. Taken together, we suggest that PWS has a high potential to treat IBD by inhibitory effect on the inflammation, and the collapse of tight junctions.

This study has some limitations. First, we focused on small-sized compounds that can undergo passive membrane transport because many criteria of physicochemical properties to determine good drug-likeness recommend molecular weight below approximately 500 Da [11,54,55]. The analysis of large molecules is more complicated than that of small molecules to simulate drug-likeness with the consideration of transporters, carriers, or metabolites of large compounds. However, some large molecules have the potential to show medicinal effects, and further research is needed to clarify their mechanism of action in PWS. Second, there is still a chance of pharmaceutical effects even if a compound violates “the rule of five” one or more times. Many approved drugs, including non-oral drugs, still exist that violate two or three criteria of the “the rule of five” that are natural products or their derivatives [56]. For example, eleutheroside A, also known as daucosterol, violates “the rule of five” because of its high lipophilicity, but prevents inflammatory responses in a dextran sulfate sodium-induced mouse colitis model [57]. Moreover, disogenin alleviated the release of inflammatory cytokines and histopathological severity in a 2,4,6-trinitrobenzene-sulphonic acid model [58]. Therefore, it should also be considered that the therapeutic effect against IBD can still be expected in compounds that violate “the rule of five”.

In conclusion, we investigated the active compounds and candidate pathways of PWS involved in IBD using network pharmacology and molecular docking methods. As a result, 26 target proteins of PWS were identified and TNF signaling pathway and apoptosis were suggested as the highly enriched mechanism in the effect of PWS against IBD. Molecular docking simulation supports network and enrichment analysis through the showing stable binding affinity of active compounds to core target proteins. We suggest that β-eudesmol and (3R,6R,7S)-1,10-bisaboladien-3-ol in the PWS exhibiting the inhibitory effect to TNF and MMP9, could be a potential mechanism for ameliorating IBD. With further experimental validation, the results of this study will provide insight and evidence into the treatment of IBD in the future.

## Figures and Tables

**Table 1:** Centrality analysis of 26 candidate target proteins of Pyeongwi-san related to inflammatory bowel disease.

Name	Degree Centrality	Eigenvector Centrality	Betweenness Centrality	Closeness Centrality
TNF (Tumor Necrosis Factor)	14	0.544	293.2	0.185
CASP3 (Caspase 3)	6	0.353	30.5	0.175
MMP9 (Matrix Metallopeptidase 9)	6	0.289	66.4	0.171
PIK3CA (Phosphatidylinositol-4,5-Bisphosphate 3-Kinase Catalytic Subunit Alpha)	5	0.091	76.7	0.157
MMP2 (Matrix Metallopeptidase 2)	4	0.217	27.7	0.169
XIAP (X-Linked Inhibitor Of Apoptosis)	4	0.274	0.0	0.164
CASP8 (Caspase 8)	4	0.274	0.0	0.164
RIPK1 (Receptor Interacting Serine/Threonine Kinase 1)	4	0.274	0.0	0.164
MYC (MYC Proto-Oncogene, BHLH Transcription Factor)	4	0.204	75.7	0.171
MMP1 (Matrix Metallopeptidase 1)	4	0.217	13.9	0.168
ESR1 (Estrogen Receptor 1)	3	0.084	0.7	0.155
NR3C1 (Nuclear Receptor Subfamily 3 Group C Member 1)	3	0.137	52.3	0.170
REN (Renin)	3	0.153	1.0	0.163
PLAU (Plasminogen Activator, Urokinase)	3	0.099	40.0	0.153
VDR (Vitamin D Receptor)	2	0.132	0.0	0.162
PIK3CD (Phosphatidylinositol-4,5-Bisphosphate 3-Kinase Catalytic Subunit Delta)	2	0.023	0.0	0.140
ELANE (Elastase, Neutrophil Expressed)	2	0.096	0.0	0.152
ACE (Angiotensin I Converting Enzyme)	2	0.132	0.0	0.162
JAK2 (Janus Kinase 2)	2	0.023	0.0	0.140
ADAM17 (ADAM Metallopeptidase Domain 17)	1	0.103	0.0	0.161
F2 (Coagulation Factor II, Thrombin)	1	0.019	0.0	0.137
LRRK2 (Leucine Rich Repeat Kinase 2)	1	0.000	0.0	0.040
MMP13 (Matrix Metallopeptidase 13)	1	0.103	0.0	0.161
SNCA (Synuclein Alpha)	1	0.000	0.0	0.040
SLC6A4 (Solute Carrier Family 6 Member 4)	0	0.000	0.0	0.038
ALOX5 (Arachidonate 5-Lipoxygenase)	0	0.000	0.0	0.038

**Table 2:** Representative molecular docking simulation results of active compounds in PWS with target proteins.

Medicine	Compound	PubChem ID	Target Protein (PDB ID)	Binding Affinity (kcal/mol)
ZR	β-eudesmol	91457	TNF (7JRA)	−8.259
ZR	(3R,6R,7S)-1,10-bisaboladien-3-ol	71813358	MMP9 (4WZV)	−8.131
GR	Glabrocoumarin	11427657	CASP3 (2C2M)	−8.238

## Data Availability

The original contributions presented in this study are included in the article/supplementary material, and further inquiries can be directed to the corresponding author.

## Supplementary Materials

The following supporting information can be downloaded at: https://www.mdpi.com/article/10.3390/biom13091322/s1, Table S1: All active compound-target pair list and target protein candidate for PWS; Table S2: Other molecular docking simulation results of active compounds in PWS on TNF, CASP3, and MMP9.
